# Large-Scale Comparison of Toxin and Antitoxins in *Listeria monocytogenes*

**DOI:** 10.3390/toxins12010029

**Published:** 2020-01-02

**Authors:** José Antonio Agüero, Hatice Akarsu, Lisandra Aguilar-Bultet, Anna Oevermann, Laurent Falquet

**Affiliations:** 1 CENSA National Center for Animal and Plant Health, San José de las Lajas Municipality 32700, Mayabeque, Cuba; jaaguero@censa.edu.cu; 2 SIB Swiss Institute of Bioinformatics, 1015 Lausanne, Switzerland; hatice.akarsuegger@vetsuisse.unibe.ch; 3 Department of Biology, UniFr University of Fribourg, 1700 Fribourg, Switzerland; 4 Vetsuisse Faculty, University of Bern, 3012 Bern, Switzerland; lisandra.aguilarbultet@usb.ch (L.A.-B.); anna.oevermann@vetsuisse.unibe.ch (A.O.); 5 USB University Hospital Basel, 4031 Basel, Switzerland

**Keywords:** toxin–antitoxin systems, *Listeria monocytogenes*, co-evolution

## Abstract

Toxin–antitoxin systems (TASs) are widely distributed in prokaryotes and encode pairs of genes involved in many bacterial biological processes and mechanisms, including pathogenesis. The TASs have not been extensively studied in *Listeria monocytogenes* (*Lm*), a pathogenic bacterium of the Firmicutes phylum causing infections in animals and humans. Using our recently published TASmania database, we focused on the known and new putative TASs in 352 *Listeria monocytogenes* genomes and identified the putative core gene TASs (cgTASs) with the Pasteur BIGSdb-Lm database and, by complementarity, the putative accessory gene TAS (acTASs). We combined the cgTASs with those of an additional 227 *L. monocytogenes* isolates from our previous studies containing metadata information. We discovered that the differences in 14 cgTAS alleles are sufficient to separate the four main lineages of *Listeria monocytogenes*. Analyzing these differences in more details, we uncovered potentially co-evolving residues in some pairs of proteins in cgTASs, probably essential for protein–protein interactions within the TAS complex.

## 1. Introduction

Toxin–antitoxin systems (TASs) were discovered because of their involvement in a biological process called post-segregational killing (PSK), a plasmid maintenance mechanism based on two plasmid-encoded genes: a toxin gene (T) and its antagonistic antitoxin (A) [1,2,3]. In this context, the toxin and antitoxin are equally distributed in the two daughter cells; however, the instability of the antitoxin will lead to an active toxin killing the cell lacking the plasmid because the antitoxin cannot be replaced. This system also could be important under some stress (e.g., antibiotic in the medium), where the toxin is released from its less stable antitoxin partner, leading to a transient metabolic shutdown and growth arrest or cell death, similar to apoptosis in higher organisms. Of particular interest, TASs have been associated with pathogenic bacterial intracellular infection and with quorum sensing [4]. In addition, pandemic bacterial strains have been shown to carry more TASs compared to non-epidemic related species [5]. 

TAS toxicity relies on various molecular mechanisms targeting diverse biological processes, e.g.*,* cell membrane integrity, assembly of the translational machinery, tRNA and mRNA stability, translation initiation and elongation steps, DNA replication, and ATP synthesis (see [6,7] for reviews). For example, the toxins MazF or PemK target and cut both free mRNA and mRNA bound to the translational machinery, while the toxin ParE targets DNA replication by inhibiting the DNA gyrase [6]. In terms of gene structure, TASs are usually found in operons with either AT or TA orientations. Those TAS operons can be acquired from mobile genetic elements such as plasmids or phages, and are also present in bacterial chromosomes [8], leading to complex heterogeneous genomic landscapes of TASs with both horizontal and vertical transmissions. 

*Listeria monocytogenes* (*Lm*) is a Gram-positive bacterium and opportunistic food-borne pathogen [9,10,11]. It is the etiological agent of listeriosis in humans and animals causing abortion, septicemia, gastroenteritis, and central nervous system (CNS) infections [12,13]. *L. monocytogenes* strains are grouped into four distinct phylogenetic lineages called I, II, III, and IV [14,15,16]. Strains belonging to lineages I and II are the most abundant isolates worldwide and particularly Lineage I is frequent in disease [16,17], while Lineage III and IV strains are very rare and predominantly isolated from (asymptomatic) animals [18]. In silico predictions using TADB2 reveal only two TASs in *L. monocytogenes* EGD-e: *lmo0113-0114* and *lmo0887-0888* [19]. There are a few studies investigating TAS systems in *L. monocytogenes* [20,21,22] focusing only on a few *L. monocytogenes* strains and a few TAS pairs. The following TAS pairs were identified mainly using in silico methods in the strain ATCC19117 (with their corresponding gene names in *L. monocytogenes* EGD-e): *lmo0113-0114*, *lmo0887-0888*, and *lmo1301-1302,* predicting their 3D structure and potential inhibitory peptides. This report also showed by qPCR that *lmo0113* is upregulated upon heat stress [21,22]. Another report investigated the TAS *lmo0887-888* of *L. monocytogenes* EGD-e in more detail, ruling out the classical MazF action, but without demonstrating the exact role of this TAS in *L. monocytogenes* [20]. They showed that *lmo0887-888* does not affect the level of persister formation upon antibiotic treatment, but the expression of σ^B^-dependent genes *opuCA* and *lmo0880* under sub-inhibitory norfloxacin treatment [20].

In this article, we first identified putative core gene TASs in a larger set of 579 *L. monocytogenes* genomes and discovered how some of these gene pairs have potentially co-evolved. In a second step using putative accessory gene TASs, we correlated our phenotypic metadata to the presence/absence of TAS genes, revealing the potential impact of specific TASs in the pathogenicity of *L. monocytogenes* strains.

## 2. Results

### 2.1. Identification of Core Gene TASs

The TASmania database [23] was queried to identify the putative TASs in the 352 *Lm* genomes that are currently in the database (list of *L. monocytogenes* genomes in TASmania, Table S1, list of TASs, Table S2). These 352 genomes were typed using the scheme cgMLST1748 at the Pasteur BIGSdb-Lm web site [24] to obtain the core gene alleles for all strains. By comparing with the TASmania hits we identified n = 14 core gene TASs (cgTASs) (Table 1) and their respective alleles (list of alleles in the 14 cgTASs, Table S3). The current knowledge on TASs in *L. monocytogenes* is rather scarce and our list included the two cgTASs already identified by TADB2 (*lmo0113-0114* and *lmo0887-0888*). TASmania extended the number of TAS candidates by one order of magnitude in all *L. monocytogenes* strains, but only a subset of them consists of core genes according to cgMLST1748.

Out of these 14 cgTASs, two cgTASs appear as orphans (*lmo0168* and *lmo0887*). Their genetic environment was studied in the annotations of the EGD-e strain [25]. In the case of *lmo0168*, no possible partner was found, as the gene is surrounded by genes located on the other strand, confirming a probable orphan antitoxin. The potential partner of *lmo0887* is *lmo0888*, which likely is a toxin mRNA interferase containing a pemK-like domain and a plasmid_toxin domain (according to TASmania), is a mazEF (according to TADB2); however, it is not identified as a core gene according to the cgMLST1748 scheme and thus must be ignored in our co-evolution analysis below. 

**Figure 1:**
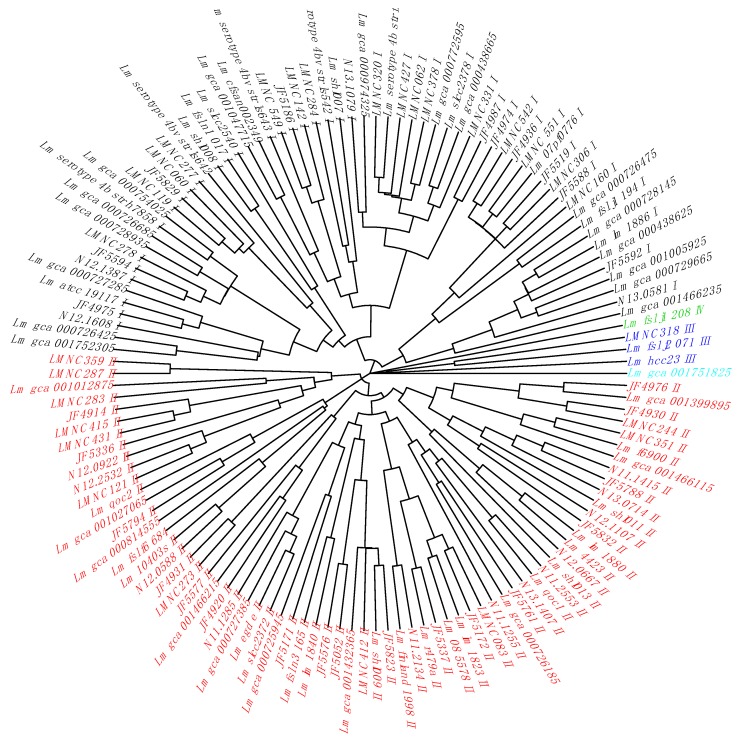
Dendrogram of the non-redundant *L. monocytogenes* strains based on the cgTAS alleles. The lineage coloration is based on the known lineages thanks to the metadata. Those without metadata are tentatively colored according to the branch. Black = Lineage I; red = Lineage II; blue = Lineage III; green = Lineage IV; light blue = *Listeria innocua* as outgroup (wrongly annotated *L. monocytogenes* in ENA).

In a second step, we added our own collection of *L. monocytogenes* strains [26] (n = 227) with their cgMLST alleles (Table S4) and metadata annotation (Table S5). We extracted their non-redundant gene alleles corresponding to the previously identified 14 cgTASs (Table S6). By clustering the non-redundant cgTAS alleles patterns with nominal hierarchical clustering, we obtained the dendrogram (Figure 1) showing that the 14 cgTASs are sufficient to separate the *L. monocytogenes* lineages (I, II, III, and IV). These results are in agreement with previous MLST analysis using seven housekeeping genes [14]. This clustering allows for characterizing the lineage membership of strains that did not have metadata information available (e.g., Lm_gca_000729665 as Lineage I and Lm_gca_001466115 as Lineage II). However, one cannot infer any causal role for the cgTASs in the lineage separation, as clustering the alleles of some subsets of core genes among the 1748 core genes would potentially show the same lineage split.

### 2.2. Co-Evolution Analysis

**Figure 2:**
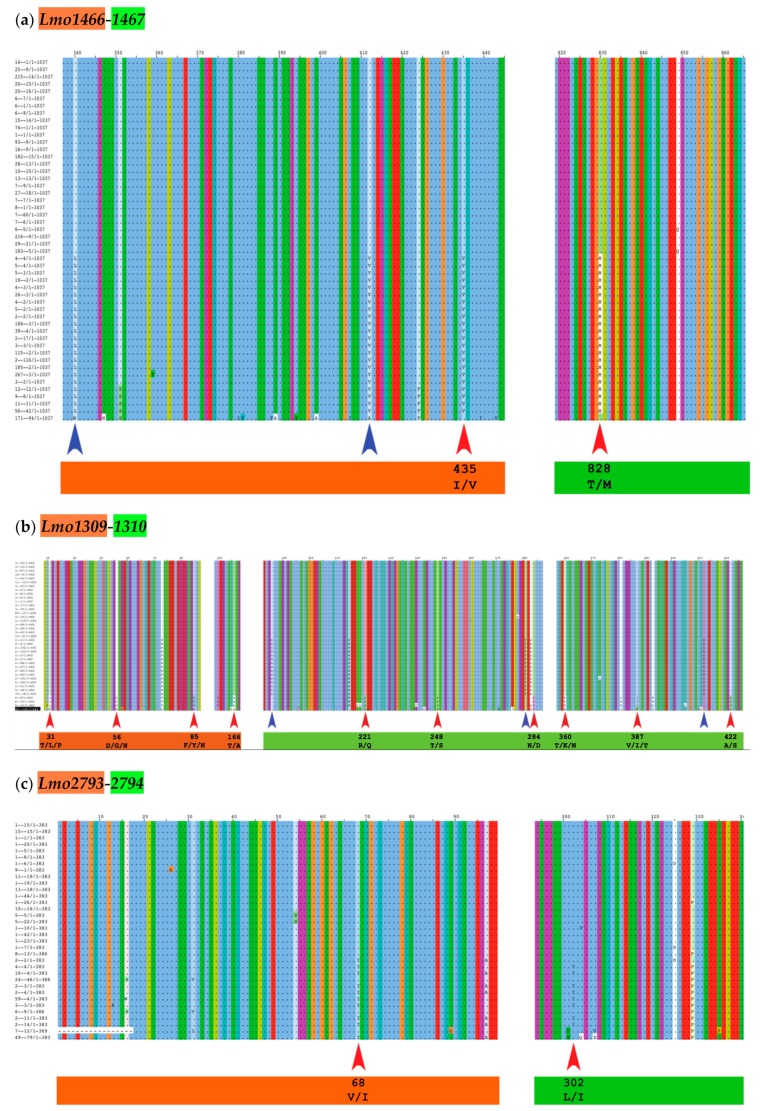
The MSA of the fusion pseudo-protein (first partner in orange, second partner in green) with the co-evolving amino acid residues highlighted (inter-protein = red arrows; intra-protein = blue arrows) within Jalview [29]. In that view, all common residues letters are hidden, only varying residues are displayed with their letters. The numbering at the top corresponds to the amino acid positions along the artificially fused protein sequence (same numbers and colors as Table 2). For clarity, only the red arrows (inter-protein) are labeled with residues highlighted in Table 2.

We analyzed the six complete cgTAS pairs for co-evolutionary residues and excluded the orphan cgTASs (*lmo0168* and *lmo0887*). We grouped the sequences by gene, translated them to proteins and fused the toxin (T) alleles with the corresponding antitoxin (A) alleles per genome in a multiple sequence alignment (MSA) using our own Perl script and MAFFT [27]. With this MSA, the BIS2Analyzer server [28] was able to identify potential co-evolutionary residues, i.e. a mutated amino acid in a toxin associated to another mutated residue in the cognate antitoxin of the same TAS (e.g., red arrows in Figure 2 and Table 2). Only two cgTAS pairs (*lmo1466-1467* and *lmo1309-1310*) revealed co-evolving residues between the two partners. For instance, the residue at location 435 (within the first partner *lmo1466*) co-evolves with the residue at location 828 (within the second partner *lmo1467*) (Figure 2a and Table 2). More than one residue per partner can be co-evolving as shown with the pair *lmo1309-1310* (Figure 2b and Table 2). One cgTAS pair (*lmo2793-2794*) had co-evolving residues visible on the MSA using Jalview (Figure 2c red arrows), but it failed to reach a significant p-value (<0.05) in BIS2Analyzer.

### 2.3. Accessory Gene TAS Analysis

**Figure 3:**
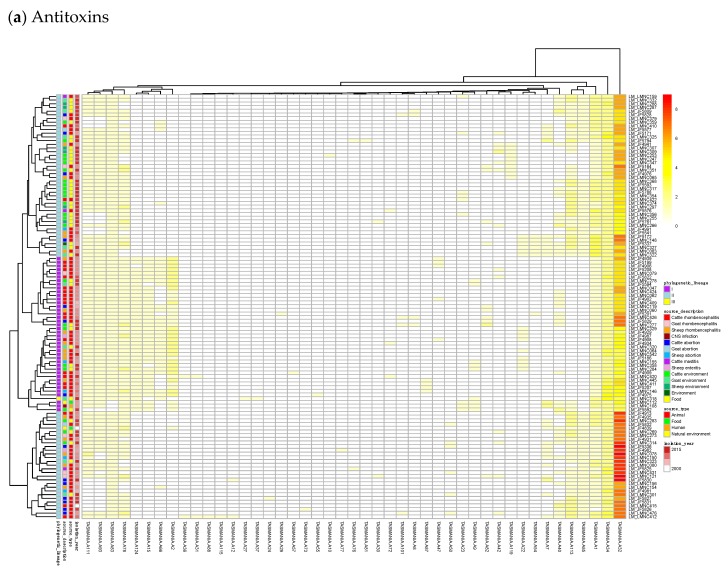

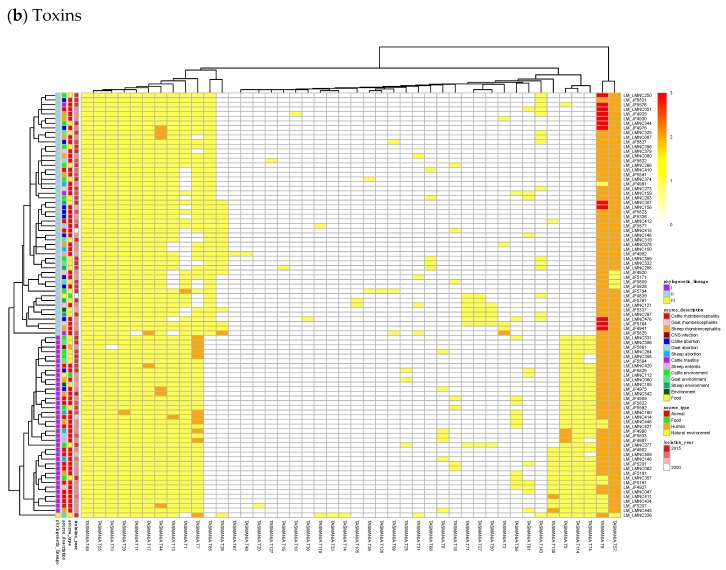
Heatmaps of the counts of accessory genes for (**a**) Antitoxins and (**b**) Toxins in 227 *L. monocytogenes* isolates. Metadata is given on the left side of the heatmap: lineage classification, source description, source type, and isolation year.

**Figure A1:**
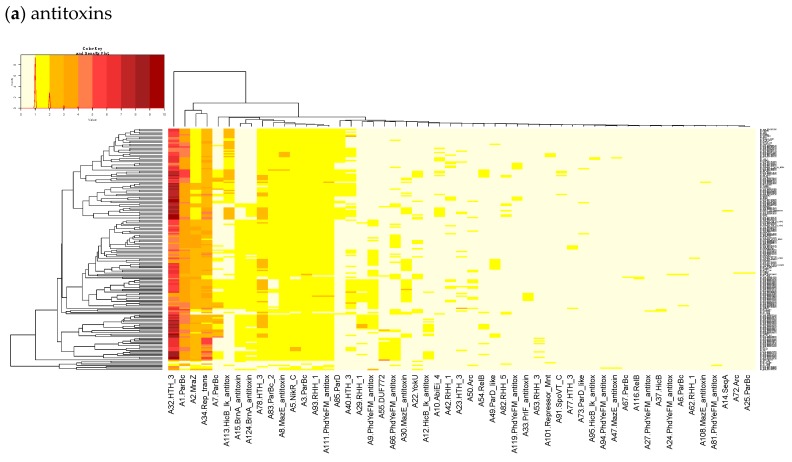

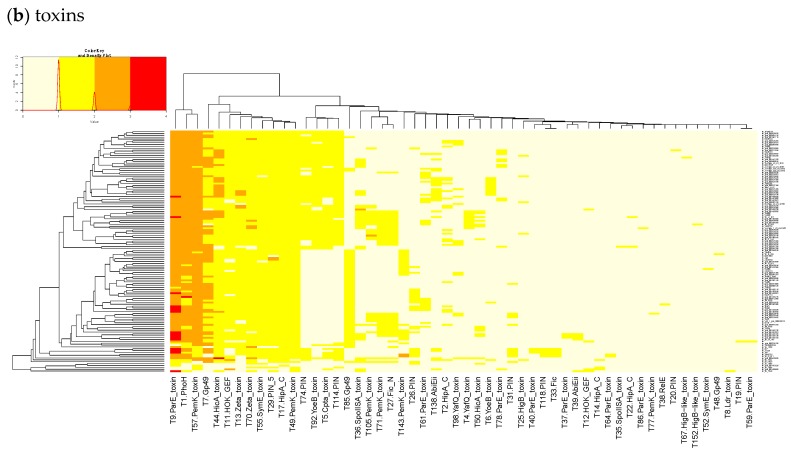
Heatmaps of the counts of accessory TAS genes (non-redundant for the strains) for (**a**) Antitoxins and (**b**) Toxins in 322 *L. monocytogenes* of TASMANIA. The closest Pfam name is given to the TASMANIA cluster.

By subtracting the cgTAS hits from the *L. monocytogenes* strains in TASmania, all other TASs are classified as accessory TASs (acTASs) (Table S7, Figure A1). In order to obtain a better understanding of the role of accessory TASs, we performed a gene analysis with a set of 227 *L. monocytogenes* strains [26] for which we have the corresponding metadata (Table S5). These isolates are not part of the TASmania database, so we processed them by first predicting protein genes with Prodigal [30] and annotating them with the TASmania HMMs [23]. Finally, we built a heatmap of those acTASs (Table S8) for antitoxins (Figure 3a) and toxins (Figure 3b) after removing the core genes and the redundancy as described above.

Figure 3a shows on the rightmost column that the antitoxin cluster A32 (HTH_3) is the most abundant and is more frequently found in animal cases of Lineage II. In TASmania, this A32 antitoxin cluster is observed as being paired with at least six different toxin clusters (T2, T13, T14, T38, T48, and T85, whose nearest Pfam identifiers are HipA_C, Zeta_toxin, HipA_C, RelE, Gp49 and Gp49, respectively). All of these pairs were analyzed for their co-occurrence in the *L. monocytogenes* strains above, but no candidate pair seems to exist in Lineage I, while only A32.T2 (nearest Pfam HTH_3.HipA_C) can be found rarely in Lineage II (Table A2). 

In Figure 3b, a small, interesting group of isolates carry one or two genes having a hit to the T138 (nearest Pfam AbiEii) toxin cluster. It is mainly found in Lineage I isolates causing rhombencephalitis, 12 out of 15 (9 in cattle, 2 in goat, and 1 in sheep). The three remaining isolates are not rhombencephalitis, but could be related by their proximity to animals: 1 sheep abortion and 2 environmental isolates. In addition, one case carrying T138 in Lineage II, a cattle rhombencephalitis, is reported. This renders this toxin quite interesting regarding rhombencephalitis. However, in the *L. monocytogenes* strains of TASmania, no antitoxin partner is known for this toxin that seems to be often encoded in a prophage region.

For the 352 *L. monocytogenes* strains described in TASmania unfortunately, no metadata is available, but the list of acTASs and their abundance is similar to our annotated dataset, with A32, A1, A2, and A34 (nearest Pfam HTH_3, ParBc, MraZ, and Omega_Repress/Rep_trans, respectively) being the most prevalent antitoxin clusters, and T9, T1, T57, T7, and T44 (nearest Pfam ParE, PhoH, PemK_toxin, Gp49, and HicA_toxin, respectively) being the main toxin clusters, as shown in Figure A1.

**Figure A2:**
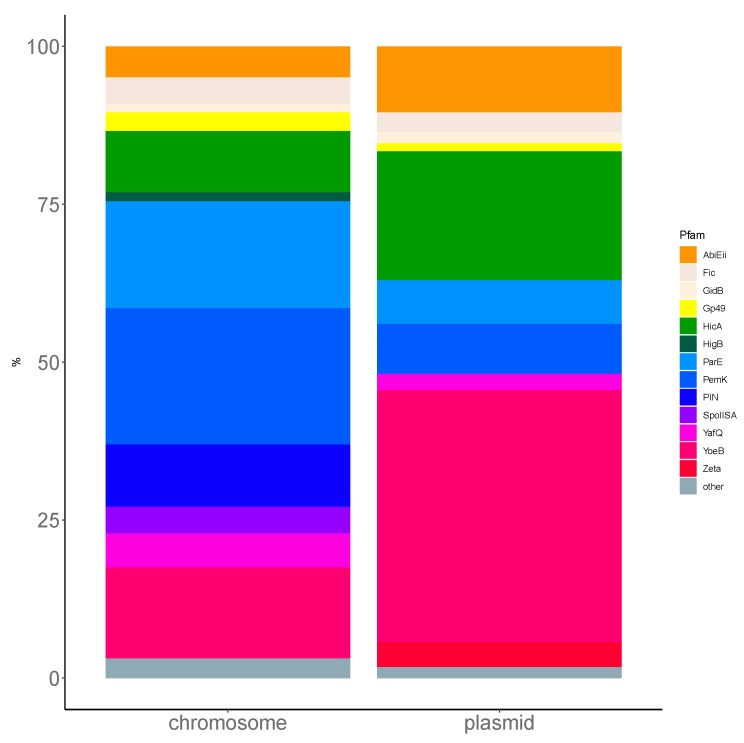
Comparison of the *Firmicutes* toxin closest to the Pfam family distribution between plasmid and chromosome. For simplicity, only TASs from the non-ambiguous two-gene AT/TA pseudo-operons were analyzed. The xT/Tx or Ax/xA pairs were ignored. *L. monocytogenes* strains, as *Firmicutes* in general, present a high prevalence of PemK and ParE-like chromosomal toxins, while YoeB and HicA toxins are more prevalent in plasmids.

Using TASmania, a closer look at the Pfam annotation of the toxin clusters highlights the diversity of the TA systems uncovered in *L. monocytogenes*. Indeed, ParE targets the DNA gyrase and, along with Gp49, belongs to the Pfam clan called “plasmid_antitox CL0136”, whose members are originally described as plasmid-encoded TASs involved in plasmid maintenance. The PhOH domain is found in cytoplasmic proteins predicted as ATPase and which are induced by phosphate starvation [31]. PemK toxins belong to the Pfam superfamily of CcdB/PemK (CL0624) known as growth inhibitors that can bind to their own promoter and act also as endonucleases. HicA is an mRNA interferase that binds to target mRNA potentially in a translation-independent manner. The *Firmicutes* phylum has a prevalence of ParE and PemK like toxins in chromosomal TASs, which correspond to the prevalence in *L. monocytogenes* (Figure A2). More experimental investigation is required to confirm these putative TASs and to understand the conditions that regulate their expression in *L. monocytogenes* of various pathogenicity.

## 3. Discussion

Toxins and antitoxins have many roles in the bacterial cells by targeting a broad range of biological processes. Their role in pathogenic bacteria involves plasmid and pathogenicity island maintenance or biofilm formation [4]. We previously stated [23] that the TASs identified in the TASmania database are putative TASs predicted purely in silico by computational means and would benefit from in vivo validation. This is also the case in this report; all TASs that we identified in *L. monocytogenes* are candidates to be confirmed experimentally.

We obtained those candidates by combining large-scale database searches leveraging on the “guilt-by-association” neighborhood criteria (“guilt-by-association” refers to potential T or A proteins identified only by their neighborhood to a hit in TASmania) with the available metadata. 

By looking at *L. monocytogenes* core genes, we identified 14 putative cgTASs that were analyzed for potential co-evolving residues. In addition, clustering their alleles confirmed the *L. monocytogenes* lineage separation. However, this lineage split is not necessarily due to the presence of these cgTASs. One of the cgTASs (*lmo0168*) appears as a putative orphan antitoxin (nearest Pfam “MazE_antitoxin”). Whether this putative antitoxin is expressed and functional remains unclear. If expressed, one could speculate about an interaction in trans with the toxin of another TAS. 

Since we unraveled the possibility of co-evolving residues, further work is needed to demonstrate these hypothetical protein–protein contacts between those residues. Interestingly, co-evolution was reported for a Type III toxin between amino acid residues of the toxin CptIN and nucleic acids of its cognate antitoxin ncRNA [32]. An intriguing gene is *lmo0888,* which is recognized as part of a TAS together with *lmo0887*, but it is not defined as a core gene contrary to its partner *lmo0887*. It is not clear to us why this happens, as, intuitively, the whole pair should be part of the core genes. One possible explanation could be that this gene is found in multiple copies in part of the *L. monocytogenes* genomes and thus was removed from the core genes [24].

Additional knowledge or hypotheses on TASs could be extracted, such as TAS association with diseases. For instance, when looking at the phenotypic link to acTASs, the toxin cluster AbiEii T138 is of particular interest given its frequent association with rhombencephalitis in ruminants. Even though not all strains isolated from rhombencephalitis harbored this toxin, the association of toxin T138 with ruminant rhombencephalitis was significant (*p*-value = 0.0413, *Χ*^2^ test). Looking at the TASs, no obvious partner antitoxin is found for this toxin probably because it is part of a prophage region that is usually less well annotated. Further work remains to be done to demonstrate which pathway or target is activated. The presence of this prophage should be validated for its suitability as a diagnostic tool in the surveillance of animal farms.

By using large-scale in silico analysis of toxin–antitoxin systems in *Listeria monocytogenes*, we demonstrated that knowledge could be extracted from combined genome sequences and associated metadata. This includes potential co-evolutionary residues, the detection of putative new toxin or antitoxin partners, as well as the suspected role for a specific prophage TAS in rhombencephalitis in ruminants.

## 4. Materials and Methods 

### 4.1. Data Used

Sequences were obtained from ENSEMBL.Bacteria [33] (352 *L. monocytogenes* strains chosen because they are included in our TASmania database) and from our previous work PRJEB15123, PRJEB15195 [34,35], and PRJEB22706 [26] (227 *L. monocytogenes* strains collected by our group with their associated metadata).

### 4.2. Identification of Putative TAS Genes using the TASmania Database

TAS candidates were extracted from our TASmania database https://bugfri.unibe.ch/tasmania [23] in all 352 *L. monocytogenes* strains included in the TASmania database with an R script directly querying the database. This list of candidates (Table S2) allows for the discovery of new TAS pairs. 

### 4.3. Identification of Core Gene TASs

In a first step, typing of all the 352 *L. monocytogenes* strains was performed at the BIGSdb-Lm https://bigsdb.pasteur.fr/listeria/database [24] with a full genome sequence query on the cgMLST1748 scheme, in order to identify the alleles of each of the 1748 core genes. In the second step, the candidate TAS genes of TASmania were crossed with the core genes, leading to a list of 14 core gene TASs (cgTASs) (Table 1). Core genes (cgMLST1748) are 1748 genes that were identified among all *L. monocytogenes* comparing thousands of *L. monocytogenes* genomes [24]. Accessory genes are only found in a subset of *L. monocytogenes* strains.

Another set, including 227 *L. monocytogenes* field isolates not belonging to TASmania, was added to the study. For these isolates, we knew the cgMLST profiles from a preceding study (Aguilar-Bultet, manuscript in preparation). The cgTAS genes identified above were identified in these 227 genomes by BLASTn and combined with the alleles of the 352 *L. monocytogenes* strains. From this, a matrix with allele information of all isolates, but containing only the 14 cgTAS loci previously identified from the combination TASmania-BIGSdb, was obtained.

### 4.4. Removing Redundancy and Clustering

First, the strains containing more than 50% of alleles with no hits in the 14 core gene set were removed. Second, only one genome was kept as representative for all the strains with 100% identical cgTAS allele patterns (in the 14 cgTASs).

The representative cgTAS patterns (Table S6) were clustered according to the cgMSLT allele identifiers using the Nominal Clustering *nomclust* R package [36]. The Nominal Clustering performs hierarchical cluster analysis (HCA) with objects characterized by nominal (categorical) variables with a distance measured by the Goodall 1 dissimilarity measure. The dendrogram obtained with *hclust* was then plotted with *plot* as a fan with labels colored according to the lineages (Figure 1).

### 4.5. Identification of Accessory Gene TASs

The accessory gene TASs (acTASs) were deduced by subtracting the cgTASs from the list of TASs extracted from TASmania. Redundancy among the 100% identical acTAS pattern was removed by keeping a single representative genome. The heatmaps were calculated with R package *pheatmap*, allowing for the addition of metadata (Tables S5 and S8). 

### 4.6. Identification of Potential Co-Evolving Residues

The BIS2Analyzer web site [28] was used to identify potential co-evolving residues. The input MSA for each of the 6 cgTAS pairs was built with an in-house Perl script and computed with MAFFT (File F1). The *Dimension* parameter was set to 2, allowing up to 2 exceptions on a column, and all other parameters were kept as defaults (Table 2). The residue positions and *p*-values (Fischer test) were evaluated by the CLAG score [37] and the server, respectively.

## Figures and Tables

**Table 1:** List of 14 putative cgTASs identified in TASmania *L.monocytogenes*. Gene locus names are taken from the reference EGD-e annotation (NCBI accession number NC_003210.1). The term “Guilt-by-association” refers to potential T or A proteins identified only by their neighborhood to a hit in TASmania [23]. The alternative shading is used to group the pseudo-operon TAS loci.

Core Gene Locus	Putative Type	HMM Cluster Hit	Description	TAS Pair ID and Orientation
*lm0113*	Toxin	Guilt-by-association	Peptidase_M78 domain-containing protein	1 AT
*lm0114*	Antitoxin	TASMANIA.A78	Toxin–antitoxin system, antitoxin component, Xre family	1 AT
*lmo0168*	Antitoxin	TASMANIA.A8	Orphan antitoxin mazE	2 A orphan
*lmo0887*	Antitoxin	TASMANIA.A5	CopG family ribbon-helix-helix protein	3 AT
*lmo1309*	Antitoxin	TASMANIA.A3	Chromosome partitioning protein ParB	4 TA
*lmo1310*	Toxin	Guilt-by-association	DUF3440 domain-containing protein	4 TA
*lmo1466*	Antitoxin	Guilt-by-association	Cyclic-di-AMP phosphodiesterase PgpH	5 TA
*lmo1467*	Toxin	TASMANIA.T1	PhoH family protein	5 TA
*lmo2041*	Toxin	Guilt-by-association	Ribosomal RNA small subunit methyltransferase H	6 AT
*lmo2042*	Antitoxin	TASMANIA.A2	Transcriptional regulator MraZ	6 AT
*lmo2790*	Antitoxin	TASMANIA.A1	ParB/RepB/Spo0J family partition protein	7 TA
*lmo2791*	Toxin	Guilt-by-association	Partition protein, ParA homolog	7 TA
*lmo2793*	Toxin	Guilt-by-association	Uncharacterized protein	8 AT
*lmo2794*	Antitoxin	TASMANIA.A1	Nucleoid occlusion protein	8 AT

**Table 2:** List of residues that are inter-protein co-evolving for each pair of cgTASs according to BIS2Analyzer. Positions numbered for the fusion pseudo-protein; orange = residues in the first partner; green = residues in the second partner. See Materials and Methods for more details. (Intra-protein co-evolving residues are shown in Table A1). Add fusion protein info.

cgTAS	*p*-Value	Positions from BIS2Analyzer	Jalview Confirmed
***Lmo1466-1467***	1.126763e-14	Positions:43582828 sequences:IT22 sequences:VM	Yes inter-protein
***Lmo1309-1310***	3.039477e-06	Positions:31568536038735 sequences:TDFTV3 sequences:LGYKI1 sequence:PNMNT	Yes inter-protein
	1.215791e-05	Positions:16622124828442235 sequences:TRTNA4 sequences:AQSDS	Yes inter-protein
***Lmo2793-2794***	Not significant	Positions:6830225 sequences:VL9 sequences:II	Yes inter-protein but not significant with BIS2Analyzer.

## Supplementary Materials

The following are available online at https://www.mdpi.com/2072-6651/12/1/29/s1, Table S1: genomes, Table S2: all TAShits, Table S3, 14cg TAS Allele, Table S4: All Sample Allele, Table S5: metadata, Table S6: NonRedundant 14cgTAS TASmania BIGSdb Allele, Table S7: acTAS TASMANIA, Table S8: acTAS_additionalstrains.

## Appendix A

**Table A1:** List of residues that are significantly co-evolving intra-protein for each pair of cgTASs.

***Lmo1466-1467***	4.024153e-16	Positions:34041227 sequences:FM22 sequences:LV1 sequence:MA	intra-protein Lmo1466
	4.719742e-07	Positions:11413418623535142445 sequences:KSEVAL5 sequences:NTAISF	intra-protein Lmo1466
	4.342162e-06	Positions:14131546 sequences:NT4 sequences:SS	intra-protein Lmo1466
***Lmo1309-1310***	3.977592e-11	Positions:18628141224 sequences:VSD15 sequences:ATE	intra-protein Lmo1310

**Table A2:** List of possible A32 TAS pairs in TASMANIA (all bacteria) vs. *Lm* strains.

First in Pair	Second in Pair	Pair Present in *Lm*	Remark
A32	T13 (Zeta toxin)	No	
A32	T14 (HipA_C)	No	
A32	T2 (HipA_C)	Yes	Only found rarely in Lineage II, in animals with mastitis (cattle) or rhomboencephalitis (goat and sheep)
T85 (Gp49)	A32	No	
T48 (Gp49)	A32	No	
T38 (relE)	A32	No	
